# A Fetal-type Variant Posterior Communicating Artery and its Clinical Significance

**DOI:** 10.7759/cureus.5064

**Published:** 2019-07-02

**Authors:** Stephen Capone, Nagma Shah, Rachael R George-St Bernard

**Affiliations:** 1 Anatomy, St. George's University School of Medicine, St. George, GRD

**Keywords:** fetal-type posterior communicating artery, circle of willis, posterior cerebral artery, pcoa, hypoplastic

## Abstract

The fetal posterior communicating artery is a well-established variant of the cerebral vasculature, occurring in 4-29% of the population. This variant can provide unique challenges in the identification and treatment of cerebrovascular disease or a cerebrovascular accident. Here we present a cadaveric case showing the presence of the fetal-type posterior communicating artery with a contralateral calcified internal carotid artery and discuss the importance of understanding this embryological variant. This case provides specific and unique clinical sequelae that require treatment to be initiated while understanding the various complications that may arise.

## Introduction

Cerebral vasculature forming the circle of Willis has a number of established variants, which include the fetal and fetal-type posterior communicating arteries (PComA). A complete circle is only found in approximately 20% of patients [1]. The fetal-type and true fetal PComA have an incidence of 4-29% and have specific considerations in both open and endovascular surgery [1]. Embryologically, this vasculature arises after 28 days, when the anterior division of the internal carotid artery (ICA) gives rise to the anterior cerebral (ACA), middle cerebral (MCA), and the anterior choroidal (AChA) arteries, and the posterior division ICA gives rise to the posterior cerebral (PCA) and posterior choroidal (PChA) arteries. The growth of the posterior circulation via the basilar followed by the vertebral arteries is stimulated by the occipital lobe and brainstem. The fetal PCA fuses medially on the distal end of the basilar and becomes the PComA whereas the PChA is incorporated into the adult PCA [2]. The most common variant is characterized by hypoplastic arteries, which are frequently found in the posterior circulation, specifically the PComA, and are commonly seen [2]. Other variants include fenestration or duplication and arterial agenesis, which primarily occur with the basilar and anterior communicating artery (AComA), respectively [2]. In these variants, the PCA is supplied by the anterior circulation from the ICA, rather than from the posterior circulation. During dissection of a donor, we noted a fetal-type PComA with severe atherosclerotic disease and suggest a unique cerebral blood supply.

## Case presentation

**Figure 1:**
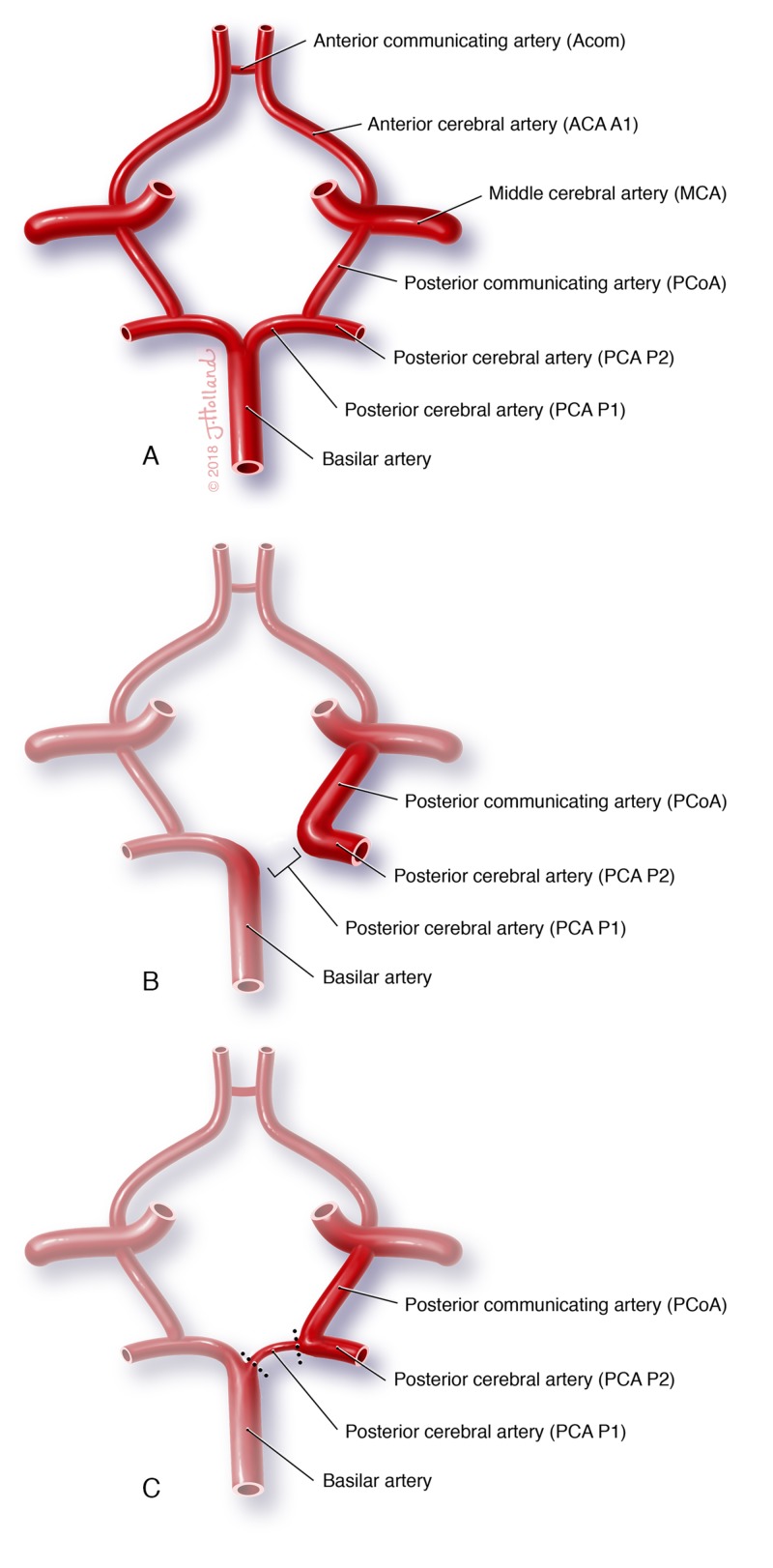
Circle of Willis Normal configuration of the A) complete Circle of Willis, noting segments of posterior cerebral artery. Abnormal configurations include B) the true fetal posterior communicating artery with an absent P1 segment and C) a fetal-type posterior communicating artery with a hypoplastic P1 segment.

A left-sided unilateral fetal-type PComA was noted during the dissection of a 66-year-old female donor. This study has been approved by St. George's University IRB (#06014). Records show the donor passed away due to congestive heart failure, suggesting the anatomic variants noted to be incidental findings. While a complete Circle of Willis is defined as the normal anatomy (Figure 1A), a fetal-type PComA is defined as having a hypoplastic P1 segment of the PCA (Figure 1C), whereas, in a true fetal PComA, the P1 segment is absent (Figure 1B). 

**Figure 2:**
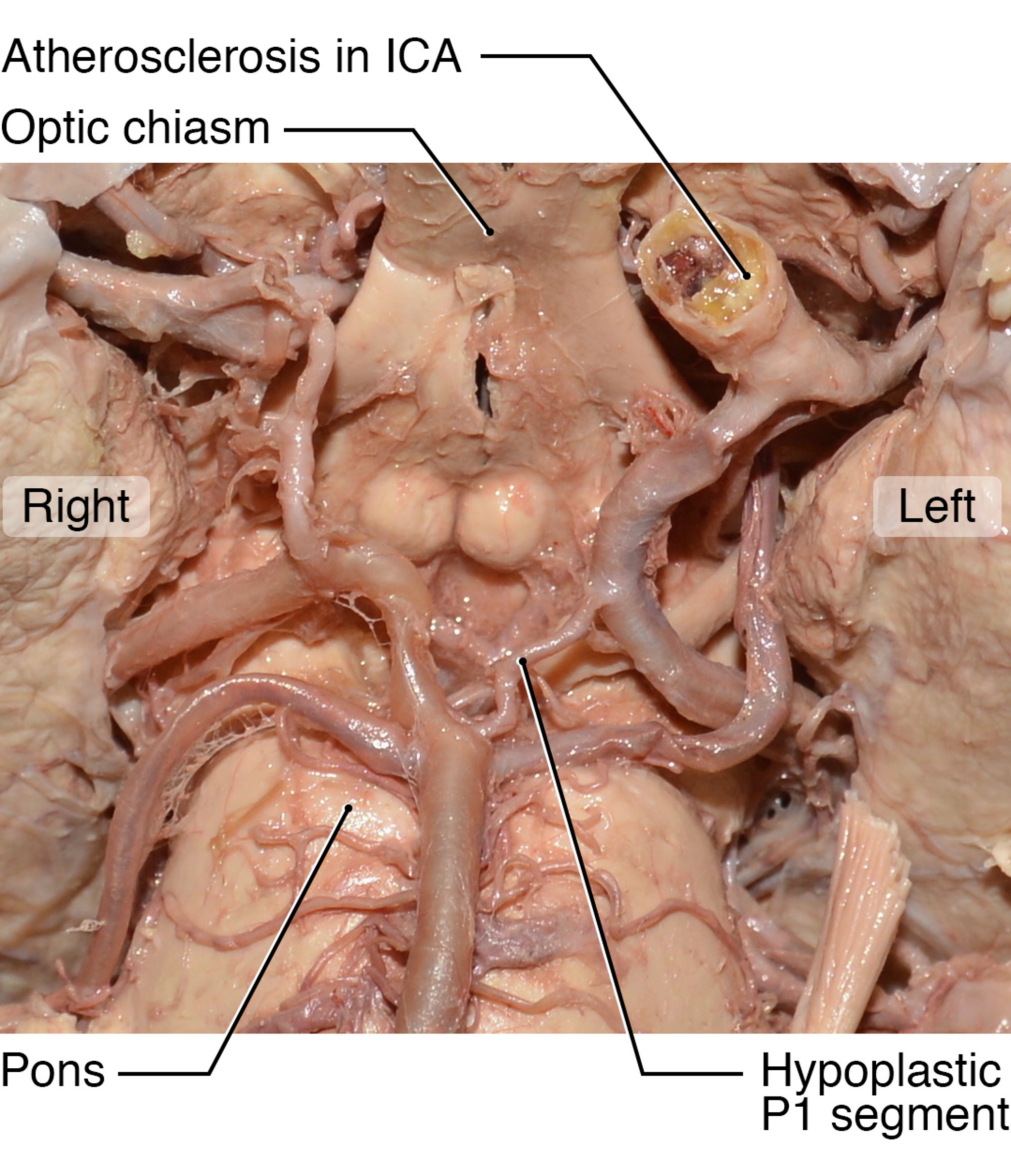
Fetal-type Posterior Communicating Artery The cadaver of a female donor with a left-sided hypoplastic posterior cerebral artery (noted) attached to a dilated posterior communicating artery. Severe atherosclerotic disease is also noted on the ipsilateral internal carotid artery (ICA).

**Figure 3:**
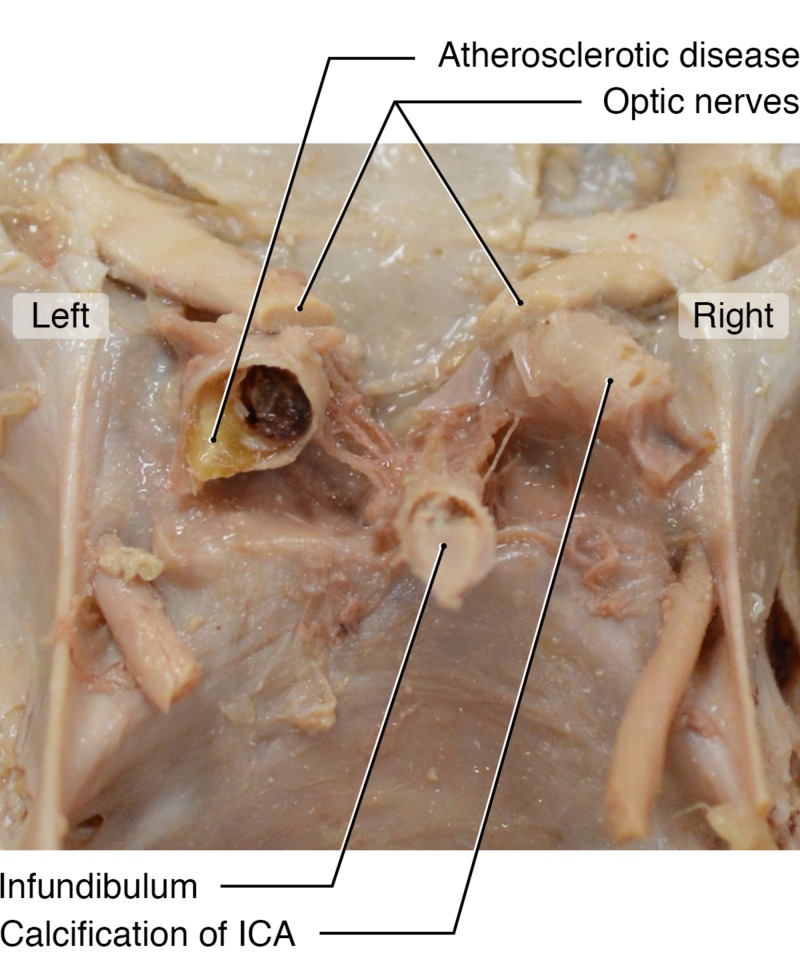
Intracranial Skull Base An intracranial view of the skull base and internal carotid arteries reveals a completely calcified and occluded right internal carotid (noted) in the same female cadaver. A stenotic and atherosclerotic left internal carotid artery is also visible.

This patient shows a large PComA directly connecting to the hypoplastic PCA (Figure 2), suggesting that the bulk of blood flow to the PCA territory was supplied by the ICA. The ipsilateral ICA was severely stenotic, with visible calcified plaque (Figure 3). Interestingly, the patient also had a completely calcified and occluded contralateral ICA (Figure 3). 

**Figure 4:**
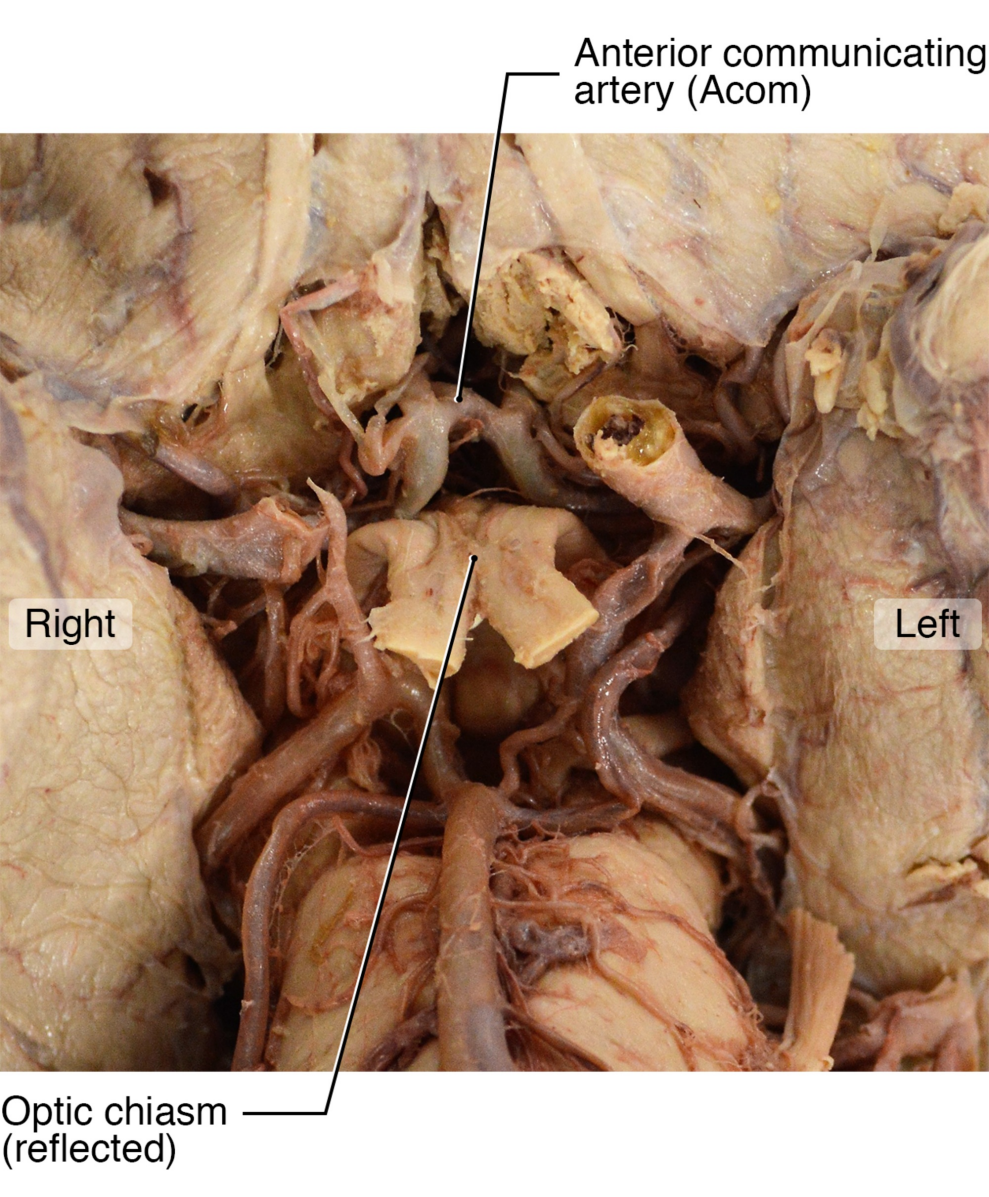
Anterior Communicating Artery A ventral view of the brainstem and circle of Willis shows the optic chiasm, reflected (noted) with a clearly visible large anterior communicating artery.

Due to these two unique variants, we assume that the vast majority of cerebral circulation was provided by the left ICA. In theory, this vessel supplied blood to five of six major cerebral vessels: the left and right ACAs, the left and right MCAs and the left PCA due to the fetal variant. We suggest that the right ACA and MCA both filled via the large-diameter AComA (Figure 4). A second possibility exists that the patient may also have had right anterior circulation filling through the vertebrobasilar system given the size of the right PComA illustrated in Figure 2.

## Discussion

These variants are of importance due to the high incidence of PComA intracranial aneurysms and the associated treatment considerations. With recent technological developments in endovascular treatments, the use of less invasive technology has been increasing. While adequate for many aneurysm subtypes, flow diverters such as the pipeline embolization device (PED) have been shown to be less effective in treating fetal-type PComA aneurysms [3-4]. Studies have shown that while stabilized with treatment, all intracranial aneurysms arising at the origin of fetal-type PComA that were treated with the PED were patent on follow-up angiographic imaging [3-4]. In these instances, the PED was positioned in the ICA in an attempt to divert the high flow from the ICA to the PComA and the PCA, which failed to work in patients with fetal-type PComA. Based on these findings, microsurgical clipping remains the optimal treatment option in patients with fetal-type PComA aneurysms. 

The fetal-type PComA has also shown difficulties in open surgery. Inadvertent injury to the fetal-type PComA during transsphenoidal surgery has been reported and can lead to severe, uncontrollable epistaxis due to the continued filling from the basilar artery [5]. It is also important to note that PComA aneurysms are properly defined as ICA-PComA aneurysms, in that they arise from the origin of the PComA at its branch point from the ICA [6]. Because of this location, the origin of the PComA is at risk for stenosis or occlusion during microsurgical clipping. An aneurysm dome with an anterolateral projection may also obscure the fetal PComA origin, and may also be adherent to the clinoid process [1]. These considerations must be carefully assessed when dissecting the surrounding tissues and mobilizing the dome for adequate exposure. When clipping PComA aneurysms, it is also important to consider the shape of the clip, as the commonly used curved clips can lead to stenosis of the PComA [6]. With a fetal variant, occlusion of the PComA is more likely to be accompanied by a significant neurological deficit. This can be due to temporary occlusion in the event of rupture or decreased patency of the vessel itself, and can manifest due to compromised occipital lobe perfusion or dependent perforator infarct [6].

Patients with fetal and fetal-type PComA may also present with paradoxical infarction patterns. The severity of anterior circulation strokes can be increased in these patients by the addition of the PCA territory, supplied by this anatomical variant [7]. Due to the multiple embryological origins that can be associated with PComA variants, the clinical significance of each is unique. True fetal PComAs are thought to be more likely to show this paradoxical infarct, as they receive no collateral blood supply from the posterior circulation [7]. In comparison, a fetal-type variant, as shown in this donor, can have compensatory filling of the PCA from the hypoplastic P1 segment of the PCA from the basilar artery, potentially alleviating some vascular insufficiency. As reported by Lambert et al (2016), this variation can be the cause of paradoxical infarct patterns or an incidental finding [7]. 

## Conclusions

This case is an example of a fetal-type posterior communicating artery, with the anterior circulation supplying the PCA via the internal carotid artery. The fetal-type PComA is also associated with increased complications in both endovascular and surgical therapy, with a unique pattern of infarcts due to the abnormal blood supply to the brain. Despite current efforts, research has been mixed and it is unclear whether the presence of a fetal or fetal-type PComA is associated with increased risk of infarction or stroke.

## References

[1] Golshani K, Ferrell A, Zomorodi A, Smith TP, Britz GW (2010). A review of the management of posterior communicating artery aneurysms in the modern era. Surg Neurol Int.

[2] Menshawi K, Mohr JP, Gutierrez J (2015). A functional perspective on the embryology and anatomy of the cerebral blood supply. J Stroke.

[3] Tsang ACO, Fung AMY, Tsang FCP, Leung GKK, Lee R, Lui WM (2015). Failure of flow diverter treatment of intracranial aneurysms related to the fetal-type posterior communicating artery. Neurointervention.

[4] Zanaty M, Chalouhi N, Starke RM, Jabbour P, Ryken KO, Bulsara KR, Hasan D (2016). Failure of the pipeline embolization device in posterior communicating artery aneurysms associated with a fetal posterior cerebral artery. Case Rep Vasc Med.

[5] Jagetia A, Rajan S, Sinha S, Singh D (2010). Fatal epistaxis from the fetal posterior communicating artery - a delayed complication of trans-sphenoidal surgery. J Clin Neurosci.

[6] Zada G, Breault J, Liu CY, Khalessi AA, Larsen DW, Teitelbaum GP, Gianotta SL (2008). Internal carotid artery aneurysms occurring at the origin of fetal variant posterior cerebral arteries: Surgical and endovascular experience. Neurosurgery.

[7] Lambert SL, Williams FJ, Oganisyan ZZ, Branch LA, Mader Jr EC (2019). Fetal-type variants of the posterior cerebral artery and concurrent infarction in the major arterial territories of the cerebral hemisphere. J Investig Med High Impact Case Rep.

